# Characterization and Optimization of L‐Asparaginase Production by *Endophytic Fusarium* sp3 Isolated From *Malcolmia aegyptiaca* of Southeast Algeria: Potential for Acrylamide Mitigation in Food Processing

**DOI:** 10.1002/fsn3.70792

**Published:** 2025-08-21

**Authors:** Wassima Lakhdari, Salah Neghmouche Nacer, Ibtissem Benyahia, Hamida Hammi, Hakim Bachir, Djawahir Mouhoubi, Yasmine Lakhdari, Ihsane Guemmou, Abderrahmene Dehliz, Barbara Sawicka, Sheikh F. Ahmad, Sabry M. Attia, Amar Djemoui, Mohammed Messaoudi

**Affiliations:** ^1^ National Institute of Agronomic Research of Algeria Station of Sidi Mehdi Touggourt Algeria; ^2^ Biology Department, Faculty of Life and Nature Sciences, Valcore Laboratory University of Boumerdes Boumerdes Algeria; ^3^ Department of Chemistry, Faculty of Exact Sciences University of El Oued El‐Oued Algeria; ^4^ Laboratory of Biogeochemistry and Desert Environments, Department of Chemistry, Faculty of Mathematics and Material Sciences University of Kasdi Merbah Ouargla Algeria; ^5^ Division of Hydraulic and Bioclimatology National Institute of Agronomic Research (INRA) Algers Algeria; ^6^ SARL SINAL Oran Algeria; ^7^ Faculty of Natural, Life and Earth Sciences Ghardaia University Ghardaia Algeria; ^8^ Department of Plant Production Technology and Commoditties Science University of Life Sciences in Lublin Lublin Poland; ^9^ Department of Pharmacology and Toxicology, College of Pharmacy King Saud University Riyadh Saudi Arabia; ^10^ Laboratory of Organic Chemistry and Natural Substances, Faculty of Exact Sciences and Computer University of Djelfa Djelfa Algeria; ^11^ Laboratoire de Recherche sur les Produits Bioactifs et Valorisation de la Biomasse, Département de Chimie, ENS Kouba Alger Algeria

**Keywords:** glucose concentration, incubation time, L‐asparaginase production, nitrogen sources, optimization, pH levels

## Abstract

This study aims to isolate and optimize the production of L‐asparaginase from fungal strains derived from Algerian Saharan plants, and evaluate the reduction of acrylamide formation in food products. L‐asparaginase has frequently been used to treat childhood acute lymphoblastic leukemia. It catalyzes the hydrolysis of asparagine and glutamine into aspartic acid and ammonia. It is also used in the food industry to minimize acrylamide formation during high‐temperature frying of starchy food items. In this study, L‐asparaginase was identified in various microbial, animal, and plant species. Using Czapek‐Dox medium, different fungal species were first isolated from Saharan plants of southeast Algeria (including *Zygophyllum cornutum* Coss., *Malcolmia aegyptiaca* Spreng., *Phoenix dactylifera* L., and *Cyperus rotundus* L.) and tested for their ability to produce extracellular L‐asparaginase. Among 13 isolates, nine were positive in the preliminary test. The strain *Fusarium* sp.₃, isolated from *M. aegyptiaca* leaves, had the highest enzyme index (1.92 ± 0.35) with maximum enzyme production (63.68 units per milliliter). Critical factors such as temperature (30°C–50°C), pH (5.0–8.0), and substrate concentration (1–10 g/L) were optimized under liquid‐state fermentation to maximize enzyme production. Utilizing Minitab software, additional statistical methods were employed for the optimization process, including the Plackett–Burman design and response surface methodology. The Plackett–Burman design screened seven variables: temperature, pH, incubation time, substrate concentration, glucose concentration, nitrogen source, and agitation speed. The design identified asparagine concentration, incubation time, and pH as the most significant factors for asparaginase production. Response surface methodology was then used to optimize these factors, producing maximum asparaginase in a 50‐mL medium. Under optimized conditions, the application of L‐asparaginase to potato slices prior to frying resulted in a 68% reduction in acrylamide content (from 435.6 ± 12.4 μg/kg to 139.3 ± 8.7 μg/kg), demonstrating the enzyme's strong potential for improving food safety.

## Graphical abstract

This study focuses on the isolation of fungal endophytes from four specific Saharan plants, in an endeavor to explore novel sources of L‐asparaginase. Diverse fungal species were isolated from Saharan flora in southeast Algeria, and using Czapek‐Dox medium, the potential to produce extracellular L‐asparaginase was evaluated. Exhibited the highest enzyme productivity, with an enzyme index of 1.92 ± 0.35 and an output of 63.68 U/mL. To enhance enzyme yield, the optimization of various physical and nutritional parameters was conducted through liquid‐state fermentation.

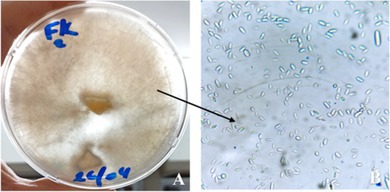

## 1 Introduction

L‐asparaginase (EC 3.5.1.1) is a crucial therapeutic enzyme that catalyzes the conversion of L‐asparagine to L‐aspartic acid and ammonia (Orabi et al. 2019). This enzyme is primarily used in cancer chemotherapy, particularly for treating acute lymphoblastic leukemia in children and adults (Vimal and Kumar 2022; Al Yousef 2022). In recent years, L‐asparaginase has also gained considerable importance in the food processing industry due to its role in mitigating acrylamide formation in thermally processed foods, thus addressing major food safety concerns (Holliday et al. 2015; Jain et al. 2012).

Acrylamide is a heat‐induced toxic compound formed during the Maillard reaction between reducing sugars and the amino acid asparagine during frying, baking, and roasting of carbohydrate‐rich foods (Pedreschi and Mariotti 2017; Shi et al. 2024). Since its identification in foodstuffs in 2002, acrylamide has raised significant health concerns, as it is classified as a probable human carcinogen (Group 2A) by the International Agency for Research on Cancer (IARC) (Ahmad et al. 2023). Numerous studies have linked dietary acrylamide exposure with increased risks of certain cancers and neurotoxicity (Upadhyay et al. 2023; Mejias et al. 2023). Therefore, the development of effective and sustainable strategies to reduce acrylamide levels in food is a priority for both industry and public health.

Enzymatic treatment with L‐asparaginase represents one of the most promising approaches for acrylamide mitigation, as it hydrolyzes L‐asparagine—the key precursor of acrylamide—without affecting food quality (Chakraborty and Shivakumar 2024; Morgan et al. 2023). The global L‐asparaginase market reached USD 380 million in 2017 and is projected to grow to USD 420 million by 2025, driven by both therapeutic and industrial applications (Parashiva et al. 2023). However, current commercial production relies predominantly on bacterial sources such as *Escherichia coli* and *Erwinia chrysanthemi* , which are associated with limitations including immunogenicity, limited stability, and challenging production processes (de Andrade et al. 2023; Muneer et al. 2020).

Fungal sources, particularly endophytic fungi, have emerged as promising alternatives for L‐asparaginase production due to their advantages in terms of enzyme stability, lower immunogenicity, and ecological adaptability (Krishnapura et al. 2016; Batool et al. 2016). Endophytic fungi, which establish symbiotic relationships within plant tissues, represent a largely untapped source of bioactive enzymes with unique properties (Mostafa et al. 2021; Awad et al. 2021). In particular, fungi associated with desert plants may exhibit superior enzyme characteristics owing to their adaptation to extreme environments (El‐Gendy et al. 2017).

Despite growing interest, there remains a significant gap in the literature regarding L‐asparaginase production from endophytic fungi associated with arid‐region plants, especially those from the Algerian Sahara. To our knowledge, no comprehensive studies have explored the potential of these local fungal strains for food‐grade L‐asparaginase production and application in acrylamide mitigation. Moreover, few studies have systematically optimized fungal L‐asparaginase production using advanced statistical approaches for industrial application.

The present study aims to address this gap by isolating and characterizing L‐asparaginase‐producing endophytic fungi from Algerian Saharan plants (*Zygophyllum cornutum Coss*., *Malcolmia aegyptiaca Spreng*., *Phoenix dactylifera L*., and *Cyperus rotundus L*.), optimizing enzyme production through Plackett–Burman design and response surface methodology, and evaluating the enzyme's efficacy in reducing acrylamide formation in a food model system. By providing novel data on the application of locally derived fungal L‐asparaginase in food processing, this research contributes to sustainable and health‐oriented enzyme production strategies with direct relevance for food safety improvement.

## 2 Materials and Methods

### 2.1 Materials and Solutions

#### 2.1.1 Plant Samples

The plant samples utilized in this study included stems and leaves of *Z. cornutum* Coss. collected from two regions in southeast Algeria: Sidi Mahdi (33° 4′18.27″N 6° 5′43.14″E) and Sidi Slimane (33°17′22.42″N 6° 5′33.16″E). Additionally, stems and roots of *C. rotundus* L. were obtained from Ouad Souf (33°22′4.12″N 6°51′5.91″E), leaves of *M. aegyptiaca* Spr. were collected from Ouad Souf, and leaves of *P. dactylifera* L. were sourced from Touggourt (33°06′00″ N 6°04′00″ E). The plant samples were collected in Mars 2022. The plants were thoroughly cleaned under running water and air‐dried before undergoing surface sterilization.

The selected plants represent a diverse set of ecological niches and traditional uses, offering a rich source of endophytic fungi with potential for producing L‐asparaginase. This approach not only highlights the sustainable use of Algeria's biodiversity but also aims to enhance the discovery of natural sources of therapeutic enzymes.

#### 2.1.2 Reagents

The following reagents and media were used throughout the experimental procedures: 70% ethanol, 2% sodium hypochlorite for surface sterilization, potato dextrose agar (PDA) for fungal cultivation, and modified Czapek Dox (MCD) agar medium, supplemented with phenol red as an indicator for L‐asparaginase activity detection. Trichloroacetic acid (TCA) and Nessler's reagent were utilized for the quantitative evaluation of L‐asparaginase production. Additionally, a 0.5 M Tris HCl buffer (pH 8.2) was prepared for enzymatic reactions, and various nitrogen sources, such as yeast extract, urea, peptone, ammonium sulfate, ammonium nitrate, and sodium nitrate, were tested for their impact on enzyme production.

### 2.2 Laboratory Equipment

The experiments utilized a variety of laboratory equipment to ensure accurate and reliable results. A centrifuge (TDZ5‐WS, Shanghai Lu Xiangyi Centrifuge Instrument Co. Ltd., Shanghai, China) was employed for separating fungal cultures from the supernatants. A spectrophotometer (752 N UV–VIS, Shanghai Metash Instruments Co. Ltd., Shanghai, China) was used to measure absorbance at 450 nm, crucial for quantifying L‐asparaginase activity. A pH meter (7110 SET 2, WTW, Xylem Analytics Germany Sales GmbH & Co. KG, Weilheim, Germany) was utilized for precise pH adjustments and measurements under various experimental conditions. The fungal cultures were incubated in a temperature‐controlled incubator (STC 3028, Shenzhen Xin Sheng S&T Co. Ltd., Shenzhen, China) set to 28°C to ensure optimal growth conditions. Sterilization of media and solutions was achieved using an autoclave (Classe‐B 23, Ningbo Hainuo Medical Instruments Co. Ltd., Ningbo, China), maintaining aseptic conditions throughout the experiments.

### 2.3 Endophytic Fungi Isolation

Plant samples were cleaned under running water and then left to air dry. Before surface sterilization, the plant material was cut into small pieces (approximately 3 cm). The sample fragments were subsequently surface sterilized by immersion in 70% ethanol for 1 min, 2% sodium hypochlorite for 3 min, 70% ethanol for 30 s, and then rinsed three times for 2 min with sterilized distilled water. The roots were aseptically placed on Petri plates containing potato dextrose agar‐based solid culture medium supplemented with penicillin (30 mg/L) to prevent bacterial growth. The roots were then incubated at 28°C until endophytic fungi began to proliferate. Emergent fungi were isolated and inoculated into the fresh potato dextrose agar antibiotic‐free medium and incubated at 28°C for 7 days. This process was repeated until a uniform, pure endophytic fungal strain could be formed and tested for its L‐asparaginase productivity (Mostafa et al. 2021). Fungal isolates were identified based on morphological characteristics using microscopic examination of colony morphology, conidial structures, and reproductive features following standard mycological identification keys and taxonomic references.

### 2.4 Determination of L‐Asparaginase Production by Endophytic Fungi

#### 2.4.1 Detection of L‐Asparaginase Activity in Fungal Isolates

To verify the production of L‐asparaginase by endophytic fungi, all isolates of endophytic fungi were cultured on Potato Dextrose Agar (PDA) for 7 days. The 5 mm disc of mycelium was transferred into the petri dishes containing Modified Czapek Dox (MCD) agar medium (glucose [2.0 g/L], L‐asparagine [10.0 g/L], KH_2_PO_4_ [1.52 g/L], KCl [0.52 g/L], MgSO_4_.7H_2_O [0.52 g/L], CuNO_3_.3H_2_O [0.001 g/L], ZnSO_4_.7H_2_O [0.001 g/L], FeSO_4_. 7H_2_O [0.001 g/L]) supplemented with phenol red (2.5% final concentration) as an indicator Control plates were MCD agar without asparagine; all the plates were incubated at 28°C. The pink zone around the colony indicates the presence of the enzyme, and the diameter of the zone was measured from positive isolates (Awad et al. 2021; El‐Gendy et al. 2017).

#### 2.4.2 Quantitative Evaluation of L‐Asparaginase Production

The fungus was cultivated for 7 days at 28°C in a modified Czapek Dox (CD) medium, and L‐asparaginase activity was evaluated using a modified technique (da Silva et al. 2022). A reaction mixture containing 0.5 mL of 0.5 M Tris HCl buffer (pH 8.2), 0.1 mL of 0.04 M L‐asparagine, 1.0 mL of adequately diluted enzyme source (culture filtrate of an endophyte), and 0.4 mL of distilled water (total volume of 2.0 mL) was incubated at 37°C for 30 min. The reaction was stopped by adding 0.5 mL of 1.5 M Trichloroacetic acid (TCA). Following the addition of TCA, blank tubes were made by adding the enzyme source. Following the completion of the reaction, 0.1 mL of the above reaction mixture was added to 3.7 mL of distilled water and 0.2 mL of Nessler's reagent, and the mixture was then incubated for 20 min. By measuring the absorbance at 450 nm, the amount of ammonia emitted during the reaction was calculated. Under assay conditions, 1 international unit (IU) of L‐asparaginase is required to liberate 1 μM of ammonia in 1 min (Equation 1) (Hashim Mohamed 2013).

(1) $$\text{Units}/{\mathrm{mL}}_{\text{enzyme}}=\frac{\left(\mathrm{\mu M}\;\text{of}\;{\mathrm{NH}}_{3\text{liberated}}\right)\left(2.5\right)}{\left(0.1\right)\;\left(30\right)\;\left(1\right)}$$

where, (2.5); initial volume of enzyme mixture (mL); (0.1); volume of enzyme mixture used in the final reaction (mL); (30); incubation time (min); (1); volume of enzyme used (mL).

### 2.5 Optimization of L‐Asparagine Production

The fungus that exhibits the higher L‐asparaginase concentration was further tested for production optimization.

#### 2.5.1 Effect of Glucose Concentrations on L‐Asparaginase Production

Three to four discs of the fungus were inoculated into Erlenmeyer flasks (250 mL) containing 50 mL of MCD liquid medium with different concentrations of glucose at 0.1%, 0.2%, 0.3%, 0.4%, and 0.5%, initial pH 6.2, and incubated at 30°C for 7 days. After incubation, the culture was centrifuged, and the supernatant was analyzed to produce the L‐asparaginase enzyme. Then, at 450 nm, the activity of the eluted L‐asparaginase was determined.

#### 2.5.2 Effect of pH

To determine the optimal pH for L‐asparaginase production, the fungal culture was introduced into a series of different pH environments, ranging from 5 to 9. pH adjustments were made using 1 N NaOH or 1 N HCl in 250 mL Erlenmeyer conical flasks containing 50 mL of the production medium. Each flask was inoculated with three to four fungal culture discs and then incubated for 7 days. Following the incubation, the culture underwent centrifugation, and the resulting supernatant was analyzed to measure the production of the L‐asparaginase enzyme. The activity of the extracted L‐asparaginase was subsequently assessed at 450 nm.

#### 2.5.3 Effect of Incubation Time

The duration required for the fungal culture to achieve peak enzyme production was established by incubating it for a period ranging from 1 to 12 days. This incubation took place in 250 mL Erlenmeyer conical flasks filled with 50 mL of the production medium, and each flask was inoculated with three to four fungal culture discs. After the incubation period, the culture underwent centrifugation, and the supernatant was analyzed to assess the production of the L‐asparaginase enzyme. The activity of the eluted L‐asparaginase was then measured at a wavelength of 450 nm.

#### 2.5.4 Effect of Nitrogen Source

Various nitrogen sources, besides L‐asparagine, were introduced and tested to enhance enzyme production. This was accomplished by adding different nitrogen sources (namely, yeast extract, urea, peptone, ammonium sulfate, ammonium nitrate, and sodium nitrate) at a concentration of 1% (w/v) to 250 mL Erlenmeyer conical flasks containing 50 mL of the production medium. Each flask was inoculated with three to four fungal culture discs and incubated for 5 days. Following the incubation, the culture underwent centrifugation, and the supernatant was analyzed to evaluate the production of the L‐asparaginase enzyme. Subsequently, the activity of the extracted L‐asparaginase was measured at a wavelength of 450 nm.

#### 2.5.5 Effect of L‐Asparagine Concentrations on L‐Asparaginase Production

Fragments culture of the fungus was inoculated into Erlenmeyer flasks (250 mL) containing 50 mL of Modified Czapek Dox (MCD) liquid medium with varying concentrations of L‐asparagine at 0.5%, 1%, 1.5%, 2%, and 2.5%; initial pH 6.2; and incubated at 30°C for 5 days. After incubation, the culture was centrifuged, and the supernatant was analyzed to produce the L‐asparaginase enzyme. Then, the L‐asparaginase activity was determined at 450 nm.

#### 2.5.6 Effect of High Nitrogen Content

The same nitrogen sources that were investigated in the previous parameter were tested; but this time, L‐asparagine was added at a concentration of 1% (w/v). This was carried out in 250 mL Erlenmeyer conical flasks containing 50 mL of the production medium. Each flask was inoculated with three to four fungal culture discs and incubated for a duration of 7 days. Following the incubation period, the culture underwent centrifugation, and the synthesis of L‐asparaginase in the supernatant was assessed. Subsequently, the activity of the extracted L‐asparaginase was measured at 450 nm.

### 2.6 Plackett–Burman

Plackett–Burman design is a great technique for selecting significant factors from a wide number of operating parameters that influence the fermentation process while requiring the least number of experimental repetitions (Yin et al. 2017). Seven independent variables in a multifactorial design were established, including Asparagine, pH, Glucose, KCl, KH_2_PO_4_, MgSO_4_, and Time (Table 1). Independent factors were studied using high (+) and low (−) levels. The mean of the data collected during each trial carried out in triplicate was interpreted as the L‐asparagine hydrolysis response. The following Equation (2) was used to calculate each variable's principal effect:

(2) $$Y\;=\;\beta_{0}\;+\;\beta_{i}\;X_{i}$$

*Y* represents the response variable (productivity or specific activity); *β*_0_ is the model intercept, and *β*_*i*_ represents the variable estimates.

**TABLE 1:** Optimization study parameters from low to high level.

Code	Variables	Levels	
		−1	1
A	pH	4	7
B	Glucose (g/mL)	1	4
C	Asparagine (g/mL)	5	20
D	KCl (g/mL)	0.32	0.72
E	KH_2_PO_4_ (g/mL)	0.75	1.75
F	MgSO_4_ (g/mL)	0.32	0.75
G	Time (day)	5	10

### 2.7 Statistical Analysis

The experimental design for screening the most influencing fermentation parameters, optimization of fermentation conditions, and statistical analysis of experimental results was performed using IBM SPSS (Statistical Package for the Social Sciences) version 24.0 for Windows. IBM SPSS was used to conduct descriptive statistics, while Minitab statistical software (version 21.0) was used to analyze the experimental designs.

## 3 Results and Discussion

### 3.1 Isolation and Screening of Endophytic Fungi for L‐Asparaginase Activity

**FIGURE 1:**
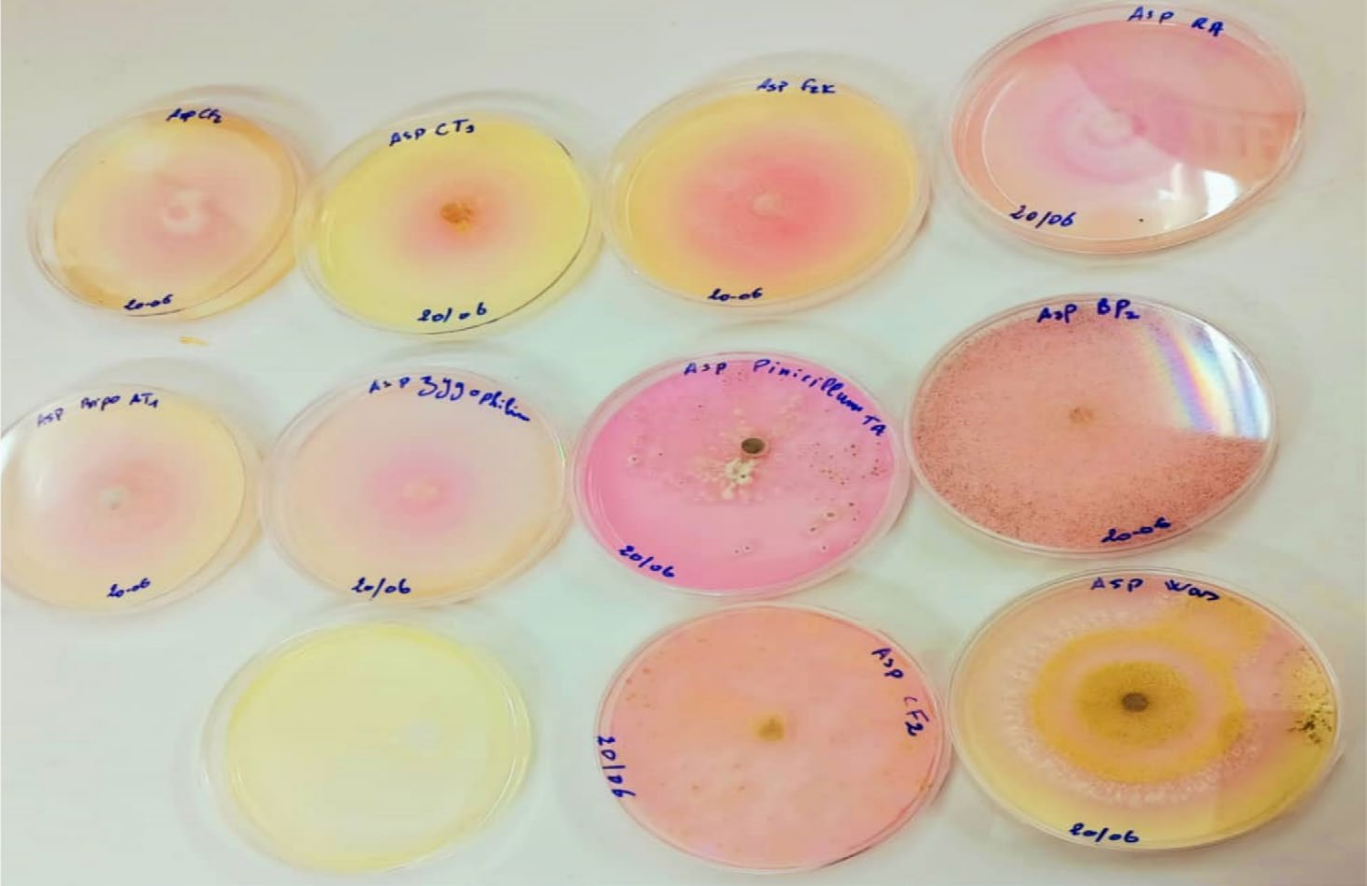
Assay for screening of L‐asparaginase production.

The experimental results showed that the plants studied from south‐eastern Algeria are rich in endophytic microorganisms. Indeed, 13 fungal isolates (Table 2) were obtained on the culture medium of potato dextrose agar. Preliminary screening of the asparaginase activity of the endophytic isolates was determined by a Petri dish test (Figure 1). Among all isolates, nine were found to be producers of L‐asparaginase (Table 3) with an enzymatic index between 1.35 ± 0.05 and 1.92 ± 0.35. However, the other four isolates were nonproducers. These nine isolates were then selected to quantitatively evaluate their L‐asparaginase production after fermentation in a liquid medium.

**TABLE 2:** Different entophytic isolates obtained from the isolation.

Endophyte isolates	Plant	Area
*Alternaria* sp	Leaves of *Zygophyllum cornutum Coss*.	Sidi Slimane
*Aspergillus flavus*	Stems of *Z. cornutum Coss*.	Sidi Mahdi
*Aspergillus niger*	Leaves of *Malcolmia aegyptiaca Spr*.	Ouad Souf
*Aspergillus terreus*	Leaves of *Phoenix dactylifera L*.	Touggourt
*Bipolaris* sp	Stems of *Z. cornutum Coss*.	Sidi Mahdi
*Chaetomium sp*	Leaves of *Cyperus rotundus L*.	Ouad Souf
*Fusarium* sp_1_	Leaves of *Z. cornutum Coss*.	Sidi Slimane
*Fusarium* sp_2_	Roots of *C. rotundus L*.	Ouad Souf
*Fusarium* sp_3_	Leaves of *M. aegyptiaca Spr*.	Ouad Souf
*Penicillium* sp	Leaves of *P. dactylifera L*.	Touggourt
*Trichoderma harzianum*_1_	Leaves of *M. aegyptiaca Spr*.	Ouad Souf
*Trichoderma harzianum*_2_	Leaves of *P. dactylifera L*.	Touggourt
*Trichoderma harzianum*_3_	Roots of *C. rotundus L*.	Ouad Souf

**TABLE 3:** Qualitative assessment of L‐asparaginase production.

Endophyte isolates	Enzyme index
*Fusarium* sp_2_	0.89 ± 0.16
*Trichoderma harzianum*_1_	1.15 ± 0.19
*Aspergillus flavus*	1.79 ± 0.84
*Fusarium* sp_3_	1.92 ± 0.35
*Penicillium* sp.	1.7 ± 0.89
*Bipolaris* sp	1.87 ± 0.08
*Trichoderma harzianum*_2_	0.88 ± 0.12
*Chaetomium* sp	NA
*Alternaria* sp	NA
*Aspergillus terreus*	0.92 ± 0.07
*Trichoderma harzianum*_3_	NA
*Aspergillus niger*	NA
*Fusarium* sp_1_	1.35 ± 0.05

Abbreviation: NA, not active.

### 3.2 Quantitative Estimation of L‐Asparaginase Production by Nesslerization

The nine positive endophyte isolates in the preliminary screening were subsequently tested quantitatively for L‐asparaginase activity. As shown in Table 4, the highest L‐asparaginase producers were, in fact, the endophytes *Fusarium* sp3. Table 4 shows that isolated from *M. aegyptiaca* Spr leaves are *Fusarium* sp3 and *Bipolaris* sp isolated from *Zygophyllum cornutum* Coss. stems with 27.84 and 20.19 IU mL^−1^ respectively. In contrast, the *fungus Trichoderma harzianum* isolated from the leaves of *P. dactylifera* L. showed the lowest activity of 4.14 IU mL^−1^, according to the results recorded in Table 4.

**TABLE 4:** Quantitative assessment of L‐asparaginase production.

Endophyte isolates	L‐asparaginase activity (U mL^−1^)
*Fusarium* sp_2_	5.96
*Trichoderma harzianum*_1_	9.10
*Aspergillus flavus*_1_	17.09
*Fusarium* sp_3_	27.84
*Penicillium* sp	11.11
*Bipolaris* sp	20.19
*Trichoderma harzianum*_2_	4.14
*Aspergillus flavus*_2_	8.06
*Fusarium* sp_1_	10.83

### 3.3 Optimization

**FIGURE 2:**
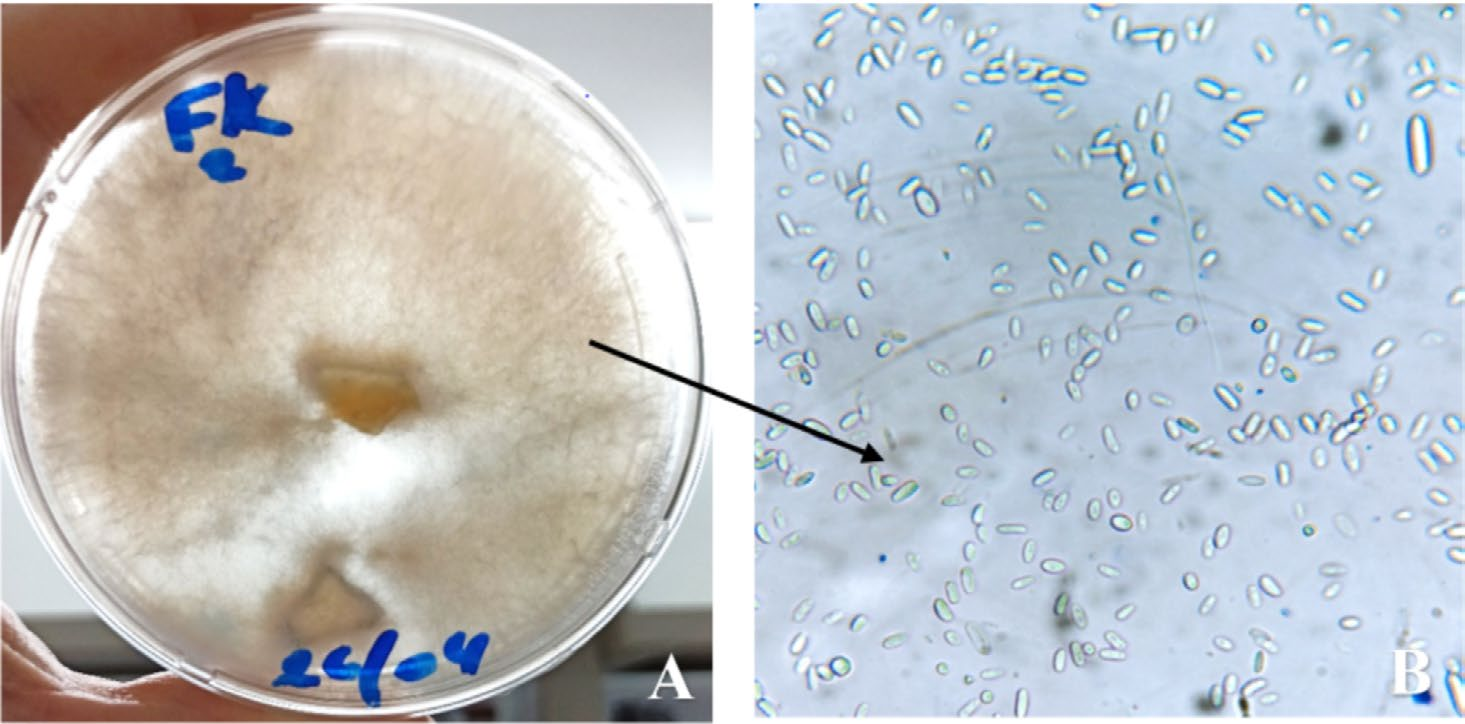
Macroscopic and microscopic aspects of *Fusarium* sp. isolate (high producer) of L‐asparaginase. (A) Macroscopic aspect. (B) Microscopic aspect.

*Fusarium* sp3 isolated from *M. aegyptiaca* leaves (the highest producer among the isolates from this plant) was morphologically identified (Figure 2) and chosen to study the effects of glucose concentrations, pH, incubation period, nitrogen source, L‐asparagine concentrations, and high nitrogen content of the culture medium on enzyme activity. Although Table 4 shows that *Fusarium* sp2, isolated from the roots of *C. rotundus* L., exhibited a high L‐asparaginase activity, our optimization study focused on Fusarium sp3 due to its higher enzyme production among isolates from *M. aegyptiaca*, which is the primary plant of interest in this study.

#### 3.3.1 Effect of Glucose Concentrations on L‐Asparaginase Production

**FIGURE 3:**
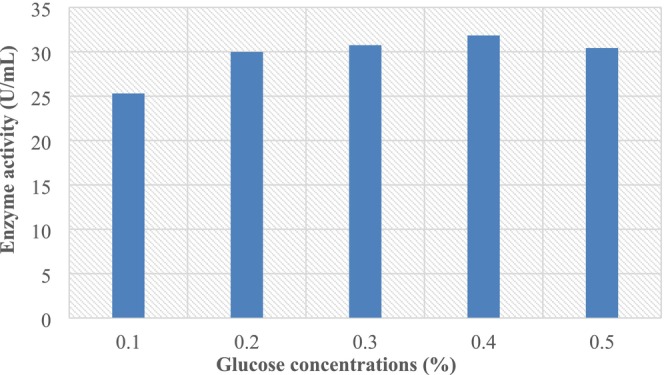
Effect of glucose concentrations on L‐asparaginase production.

To examine the effect of glucose concentration, the liquid state fermentation was carried out with different concentrations of glucose varying from 0% to 0.5%. According to data in (Figure 3), the highest L‐asparaginase activity (63.68 U/mL) was achieved with the optimum glucose concentration of 0.4%. The enzyme activity decreased as glucose concentration increased; this could be due to high glucose concentrations having an inhibitory effect.

Glucose served as the preferred carbon source for *Aspergillus terreus* CCT 7693 L‐asparaginase (Brumano et al. 2019), *F. equiseti*, *Fusarium semitectum*, and *A. niger* (Adeniran et al. 2013), *Aspergillus* sp. ALAA‐2000 (El‐Gendy et al. 2021), and *F. solani* AUMC 8615 (El‐Gendy et al. 2021; Isaac and Abu‐Tahon 2016). These might be explained by the fact that fungi grow and produce many metabolites more readily from simple sugars than from complex sugars.

The optimum level of glucose in the present study was higher than 0.3% glucose as a carbon source presented the maximum production of L asparaginase using *Fusaium oxysporum* (Meghavarnam and Janakiraman 2017). In fact, 0.4% glucose was found to be the best carbon source for maximum L‐asparaginase production by *Bipolaris* sp. isolate BR438 (Hosamani and Kaliwal 2011).

#### 3.3.2 Effect of pH

**FIGURE 4:**
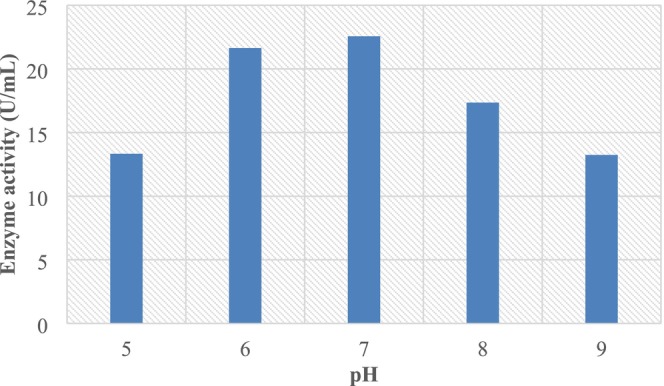
Effect of pH on L‐asparaginase production.

As indicated in (Figure 4), the initial pH values affected the peak of L‐asparaginase activity. The greatest enzyme activity (45.12 U/mL) was determined at pH 7.0, but at acidity equal to pH 5.0 or alkalinity equivalent to pH 9.0, it decreased to 26.67 U/mL and 26.48 U/mL, respectively. Since microorganisms are sensitive to the amounts of hydrogen ions present during fermentation, pH is the most important factor controlling enzyme secretions (Dong et al. 2023; Zhao et al. 2024; Delavar and Wang 2021). L‐Asparaginase from *A. terreus* CCT 7693, *A. fumigatus* , and *F. solani* AUMC 8615 exhibited the highest enzyme production (13.81, 23.83, 20.57 U mL^−1^) at pH 9.49, 8.0, and 7.0, respectively. While these studies provide relevant comparisons, our results highlight the distinct pH sensitivity observed in our local strains (El‐Gendy et al. 2021; Shenbagamuthuraman et al. 2022; Silva et al. 2022). Extreme pH is likely to result in irreversible alterations in enzymes by harming particular amino acids at the active sites at higher pH levels in addition to hydrolyzing peptide bonds at lower pH levels (El‐Gendy et al. 2021; Pallem 2019).

#### 3.3.3 Effect of the Incubation Period

**FIGURE 5:**
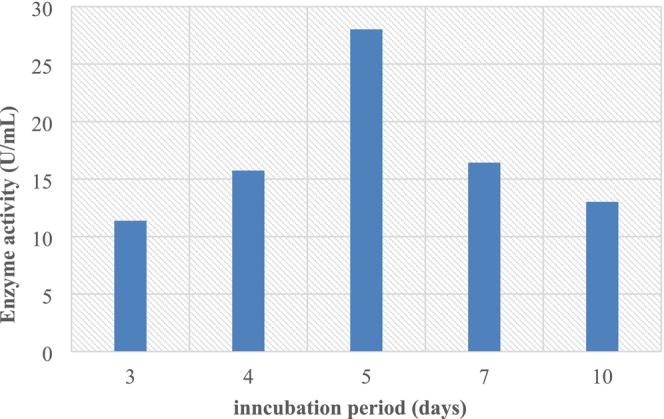
Effect of incubation period on L‐asparaginase production.

Increasing the incubation time led to a progressive improvement in L‐asparaginase activity, which peaked after 5 days at 55.31 U/mL and then decreased to 41.43 U/mL and 37.02 U/mL after 7 and 10 days of fermentation, respectively (Figure 5). Our results are in line with (Isaac and Abu‐Tahon 2016; Bhosale and As‐Suhbani 2019), who noticed that the optimal productivity of L‐asparaginase from *Fusarium oxysporum* and *Fusarium solani* AUMC 8615 occurred on Day 5 of the incubation period. Still, *Fusarium equiseti* AHMF4 secreted the maximum yield of enzyme (12.57 U mL^−1^) after a week (Pallem 2019).

**FIGURE 6:**
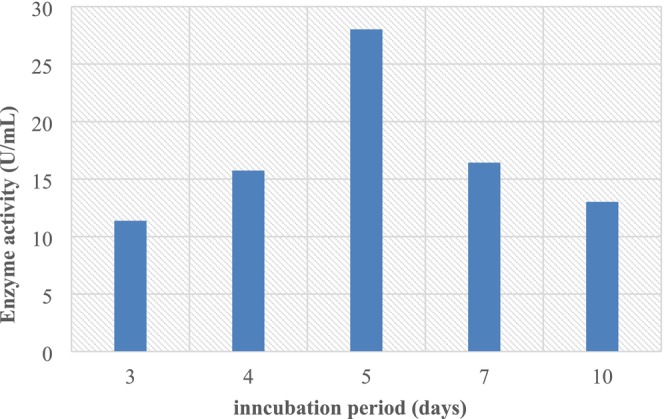
Effect of nitrogen source on L‐asparaginase production.

**FIGURE 7:**
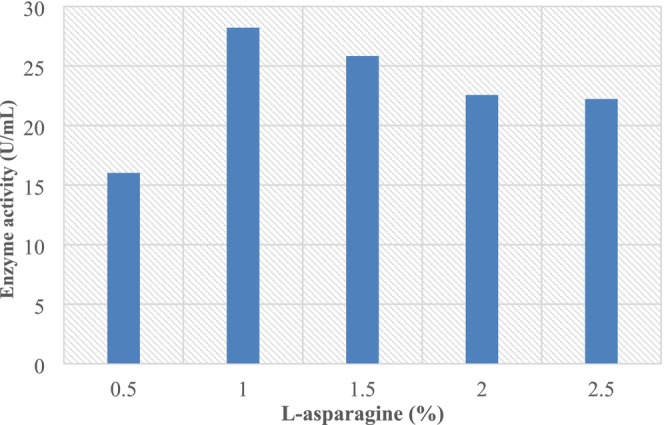
Effect of L‐asparagine concentrations on L‐asparaginase production.

**FIGURE 8:**
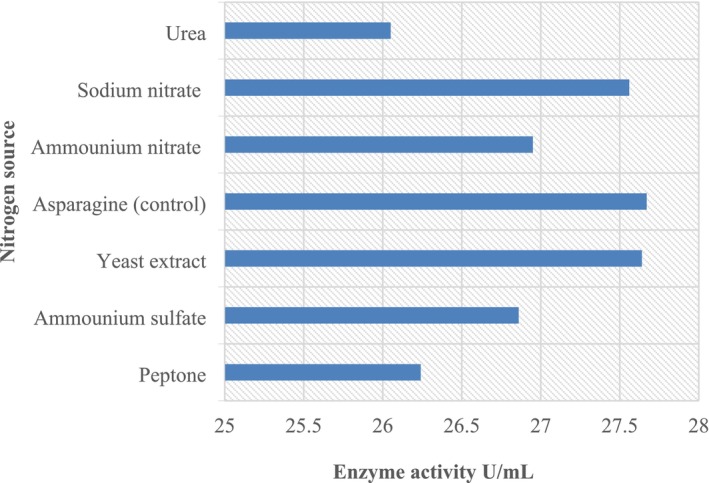
Effect of high nitrogen content on L‐asparaginase production.

In Figures 3, 4, 5, 6, 7, 8, the presented values correspond to the extracellular L‐asparaginase activity (expressed in U/mL). The numerical values mentioned in the text are directly taken from these figures to ensure clarity and consistency.

#### 3.3.4 Effect of Nitrogen Source

Data in Figure 6 presented the impact of nitrogen sources on L‐asparaginase activity. The nitrogen sources yielded 42.78, 39.57, 34.87, 29.75, 21.79, 16.42, 55.56, and 8.21 U/mL of extracellular L‐asparaginase activity, respectively. According to our results, asparagine was the best nitrogen source used to increase the production of L‐asparaginases by the endophyte *Fusarium* sp3 isolated from *M. aegyptiaca Spr* leaves, in accord with those (El‐Gendy et al. 2021; Prihanto et al. 2019; Benchamin et al. 2019) by the endophytes *Aspergillus fumigatus* and *F. oxysporum*.

#### 3.3.5 Effect of L‐Asparagine Concentrations on L‐Asparaginase Production

The effect of different concentrations of L‐asparagine (0.5%–2.5%) was further studied to maximize the production of the enzyme. According to data in (Figure 7), the highest L‐asparaginase activity (28.21 U/mL) was achieved with the optimum L‐asparagine concentration of 1%. Further increase in L‐asparagine concentration resulted in the decrease of enzyme production; it may be due to inhibitory effect at higher concentrations. The highest enzyme productivity by *Aspergillus* sp. ALAA‐2000 (39.39 U mL^−1^) and *T. asahii* IBBLA1 (20.57 U mL^−1^) was achieved with asparagine at a concentration of 1.0% (El‐Gendy et al. 2021; Ashok and Devarai 2019).

#### 3.3.6 Effect of High Nitrogen Content

The results obtained (Figure 8) did not show a great difference between the type of nitrogen sources and the L‐asparaginase activity; the values were between 56.05 and 52.10 U/mL, respectively. Our results are comparable with those of (Sarkar et al. 2014) which demonstrated no substantial variance between the type of nitrogen sources and L‐asparaginase yield obtained from *F. oxyporum* (Sarkar et al. 2014; Mangamuri et al. 2017).

### 3.4 Statistical Analysis

*Fusarium* sp^3^ exhibited the highest enzyme production among the studied fungi, reaching 63.68 U/mL, as shown in Figure 3. This strain was selected for the statistical optimization of the L‐asparaginase production process. Our previous findings demonstrated that the concentration of certain factors, such as asparagine, pH, glucose, nitrogen, yeast extract, peptone, and time, significantly affects L‐asparaginase production. Furthermore, statistical modeling can optimize the medium factors for improved L‐asparaginase production.

Our results demonstrate that nine fungi were able to produce the enzyme L‐asparaginase. The descriptive statistics presented in this analysis provide information about two variables: endophytic fungi and their concentration. The data were collected from 27 samples (Table 5). Overall, these descriptive statistics suggest that the data exhibit great variability.

**TABLE 5:** Descriptive analysis of L‐asparaginase production.

	*N*	Minimum	Maximum	Mean	Ecart type
Endophytic fungi	27	0.00	8.00	4.000	2.63138
Concentration	27	8.15	55.68	23.6512	13.88857

The results of the normality tests indicate that the data follow a normal distribution. Therefore, to analyze significant differences between data groups, parametric tests such as analysis of variance (ANOVA) are appropriate.

The ANOVA indicates a significant difference between the studied concentration groups, with an *F* value of 447.024 and *p* < 0.001 (Table 6).

**TABLE 6:** The analysis of variance (ANOVA).

	Sum of squares	Of	Medium square	*F*	Sig.
Inter‐groups	4990.086	8	623.761	447.024	0.001
Intragroup	25.117	18	1.395		
Total	5015.202	26			

These results show significant differences in the mean concentrations of different fungi. The comparison method used is Tukey's analysis with a significance level of 0.05 (Table 7). The fungi that have significantly different mean concentrations from each other are: *T. harzianum, Fusarium* sp_2_, *A. terreus, Trichoderma* sp, *Fusarium* sp_1_, *Pinicillium* sp, *Aspergillus flavus*, *Bipolaris* sp and *Fusarium* sp_3_. The other pairs of fungi did not show significant differences in their mean concentrations. The error term used for this analysis is the mean square error, which is 1.395. The means for each group were calculated using a sample size of the harmonic mean of 3.

**TABLE 7:** Tukey's analysis of fungi groups.

Endophytic fungi	*N*	Subset for alpha = 0.05					
		1	2	3	4	5	6
*Trichoderma harzianum*	3	8.2510					
*Fusarium* sp_2_	3	11.6257					
*Aspergillus terreus*	3		15.9260				
*Trichoderma* sp.	3		18.2923	18.2923			
*Fusarium* sp_1_	3			20.3090			
*Pinicillium* sp.	3			20.8027			
*Aspergillus flavus*	3				24.2797		
*Bipolaris* sp.	3					39.1973	
*Fusarium* sp_3_	3						54.1770
Sig.		0.050	0.315	0.251	1.000	1.000	1.000

*Note:* The group means of the homogeneous subsets are displayed.

### 3.5 Statistic Results From Minitab

The Plackett–Burman Design is used to screen the significant factors that can affect the activity of L‐asparaginase produced by Fusarium sp3 isolated from *M. aegyptiaca*. The seven factors considered in the study are A (pH), B (Glucose), C (Asparagine), D (KCl), E (KH_2_PO_4_), F (MgSO_4_), and G (Time) (Table 8).

**TABLE 8:** Seven independent variables with coded values and the observed L‐asparaginase activity were evaluated using a 12‐trial Plackett–Burman experimental design.

Run	Blk	A	B	C	D	E	F	G	Asparagine activity (IU mL^−1^)
1	1	−	−	+	+	+	−	+	99.259
2	1	−	−	−	+	+	+	−	137.469
3	1	+	+	−	+	+	−	+	104.383
4	1	+	+	−	+	−	−	−	117.037
5	1	−	−	−	−	−	−	−	67.531
6	1	+	−	−	−	+	+	+	110
7	1	−	+	+	+	−	+	+	143.889
8	1	+	−	+	−	−	−	+	91.914
9	1	−	+	−	−	−	+	+	135.060
10	1	−	+	+	−	+	−	−	115.430
11	1	+	+	+	−	+	+	−	143.880
12	1	+	−	+	+	−	+	−	99.259

The goal of this research was to optimize the medium composition for L‐asparaginase production by the fungus *Fusarium* sp3. Seven parameters were examined to identify the ideal medium components for L‐asparaginase synthesis. Table 8 shows the L‐asparaginase activity from the 12 runs. The Plackett–Burman design was used to identify and evaluate the key variables that could affect enzyme production. The results in Table 8 show that run number 7 had the highest L‐asparaginase activity (143.889 IU mL^−1^), while run number 5 had the lowest L‐asparaginase activity (67.531 IU mL^−1^). These differences demonstrate the importance of medium optimization in increasing L‐asparaginase production.

Based on the Plackett–Burman design, the regression equation shows how the concentration of L‐asparaginase is influenced by the various independent variables (pH, Glucose, Asparagine, KCl, KH_2_PO_4_, MgSO_4_ and Time). This Equation (3) can be used to predict L‐asparaginase production under different conditions, which can be useful for optimizing the production process.

(3) $$\begin{array}{cc} Y\;=\; & 93.9-6.15\;\mathrm{pH}+3.94\;\text{Glucose}+1.627\;\text{Asparagine}\\ & -6.0\;\mathrm{KCl}-0.3\;{\mathrm{KH}}_{2}{\mathrm{PO}}_{4}+19.7\;{\text{MgSO}}_{4}+2.68\;\text{Time} \end{array}$$

*Y* represents the experimental response (enzyme production), and the coefficients represent the effect of the parameters on asparaginase production.

The statistical model used in this study indicates that the selected independent variables explain a substantial portion of the variation in L‐asparaginase production, with a coefficient of determination (*R* ^2^) of 91.99%, reflecting a strong fit of the model to the experimental data (Table 9). Multiple linear regression analysis (Table 3) identifies pH, asparagine concentration, and incubation time as significant factors influencing L‐asparaginase production (*p*‐value < 0.05).

**TABLE 9:** Factorial regression: L‐asparaginase versus Asparagine; pH; glucose; KCl; KH_2_PO_4_; MgSO_4_; time.

Term	Coef	SE coef	95% CI	*T*‐value	*p*	VIF
Constant	37.03	9.09	(11.79; 62.26)	4.07	0.015	
pH	0.168	0.868	(−2.242; 2.577)	0.19	0.019	1.00
Glucose	2.891	0.868	(0.481; 5.300)	3.33	0.856	1.00
Asparagine	1.715	0.174	(1.233; 2.197)	9.88	0.001	1.00
KCl	9.34	6.51	(−8.73; 27.41)	1.43	0.225	1.00
KH_2_PO_4_	1.77	2.60	(−5.46; 9.00)	0.68	0.534	1.00
MgSO_4_	7.23	6.51	(−10.84; 25.30)	1.11	0.329	1.00
Time	−2.381	0.521	(−3.826; −0.935)	4.57	0.010	1.00

Abbreviations: CI, confidence interval; Coef, coefficient; SE Coef, standard error of coefficient; VIF, variance inflation factor.

Table 9 shows the ANOVA for the linear model on the effect of independent variables on L‐asparaginase production from *Fusarium* sp_3_. Fisher's *F*‐test and the ANOVA of the regression model show that the model is very significant.

ANOVA for the linear model (Table 10) shows that the overall regression model is significant (*F*‐value = 1.78, *p*‐value = 0.301), meaning that the independent variables together explain a significant portion of the variation in L‐asparaginase production.

**TABLE 10:** ANOVA of asparaginase production generated by Minitab software.

Source	DF	Adj SS	Adj MS	*F*‐value	*p*
Regression	7	3969.37	567.05	1.78	0.301
pH	1	1020.12	1020.12	3.21	0.148
Glucose	1	419.86	419.86	1.32	0.314
Asparagine	1	1786.54	1786.54	5.62	0.077
KCl	1	17.38	17.38	0.05	0.827
KH_2_PO_4_	1	0.22	0.22	0.00	0.980
MgSO_4_	1	185.49	185.49	0.58	0.487
Time	1	539.77	539.77	1.70	0.262
Error	4	1270.96	317.74		
Total	11	5240.34			

Abbreviations: Adj MS, adjusted mean square; Adj SS, adjusted sum of squares; DF, degrees of freedom.

However, the ANOVA table also shows that only two of the individual independent variables, asparagine and pH, have significant effects on L‐asparaginase production (*F*‐values of 5.62 and 3.21, respectively, with *p*‐values of 0.077 and 0.148, respectively).

The results of this regression analysis suggest that asparagine is a critical factor for L‐asparaginase production. This information could be used to optimize the production of L‐asparaginase by ensuring that the medium contains a sufficient concentration of asparagine.

Additionally, the results suggest that L‐asparaginase production decreases over time. This information could be used to develop strategies to increase the yield of L‐asparaginase, such as harvesting the enzyme at regular intervals or adding nutrients to the medium to extend the production period.

**FIGURE 9:**
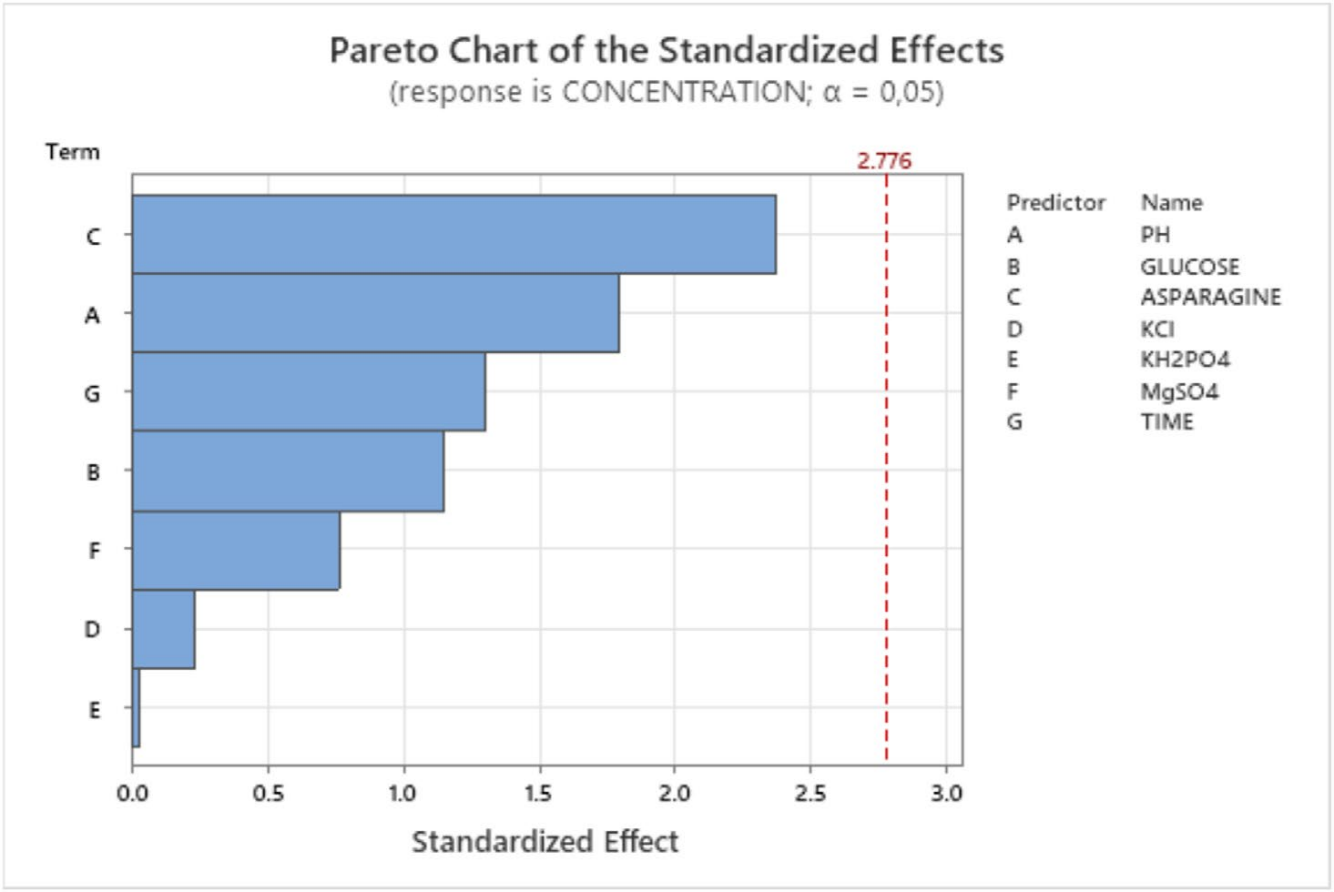
Pareto chart illustrates the order of significance of the variables affecting L‐asparaginase production.

The Pareto chart of the effects (Figure 9) shows the relative importance and order of the factors influencing L‐asparaginase synthesis. The chart shows that the elements crucial for L‐asparaginase production are Asparagine, pH, and Glucose.

**FIGURE 10:**
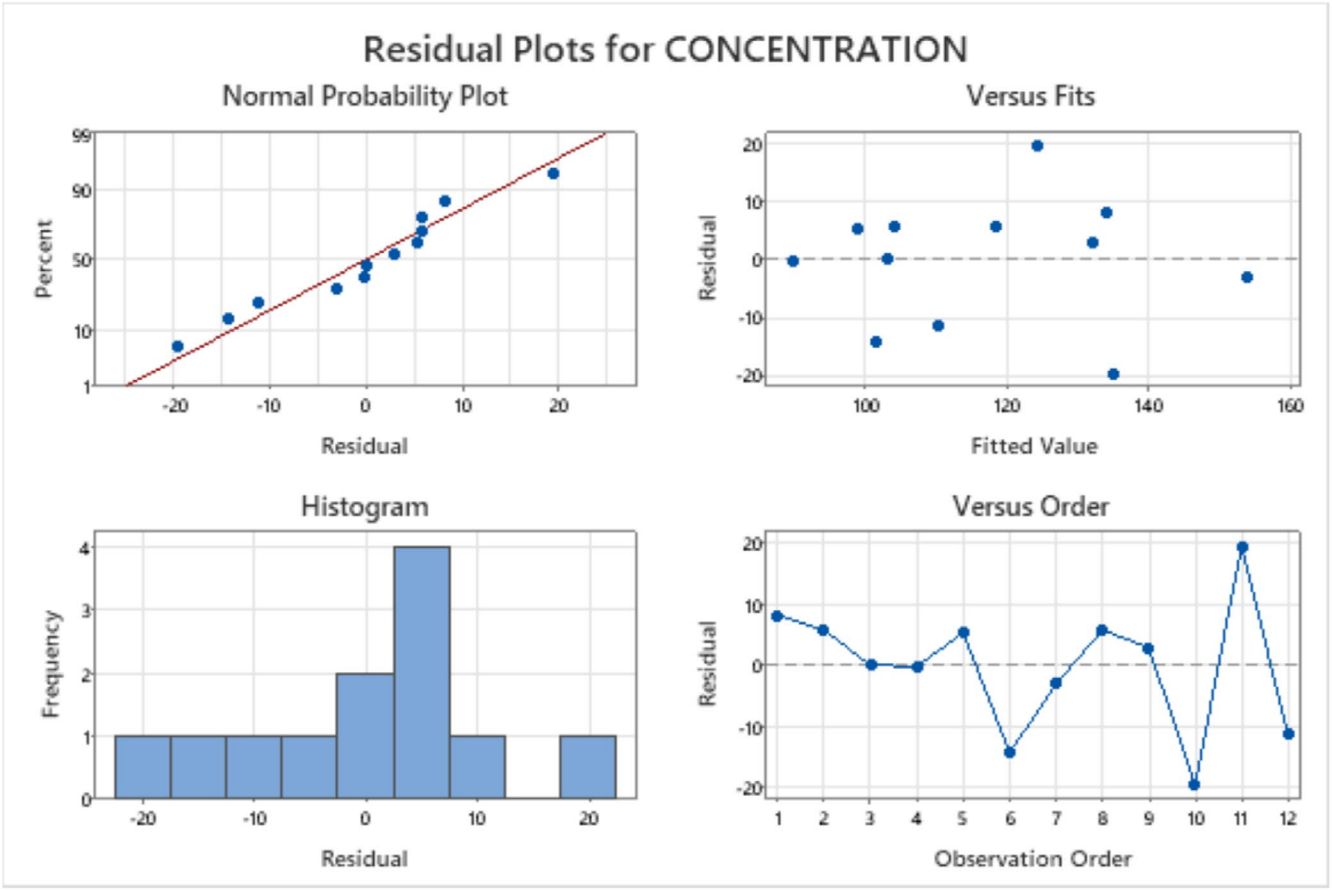
Normal probability plot of residuals for L‐asparaginase production.

The residuals' normal probability plot (Figure 10) is a crucial diagnostic tool for determining and clarifying consistent departures from the presumptions. The residual plots suggest that the linear regression model is a reasonable fit to the data. However, there are some minor violations of the assumptions of the regression model.

The optimization of parameters and statistical techniques was carried out in two stages. Firstly, the Plackett–Burman design was used to screen different operating parameters, and significant variables were identified. Secondly, the Box–Behnken method (Table 11) was used to investigate the interactions between these significant parameters. Finally, the significant variables were further optimized using Response Surface Methodology (RSM). The statistical technique RSM is used to optimize process parameters by examining the effects of different independent variables on a measurable response (Table 13). In this case, the three independent variables are pH, glucose, and asparagine, and the measurable response is not mentioned in the given Table 12. Analyzing the data from these 15 experiments will allow for modeling the relationship between the independent variables and the response. From Tables 6 and 7, the model *F* value and *p* value were found significant. Given that *p* < 0.05 was taken into consideration, the model Equation (4) is significant.

(4) $$\begin{array}{cc} Y\;=\; & -35.19+16.32\;\mathrm{pH}+0.7119\;\text{Glucose}+0.1607\;\text{Asparagine}\\ & -1.232\;{\mathrm{pH}}^{2}+0.002963\;{\text{Glucose}}^{2}+0.04347\;{\text{Asparagine}}^{2}\\ & +0.000000\;\mathrm{pH}\times \text{Glucose}-0.000000\;\mathrm{pH}\times \text{Asparagine}\\ & -0.000000\;\text{Glucose}\times \text{Asparagine} \end{array}$$

**TABLE 11:** Box–Behnken design and results for asparaginase production.

Term	Coef	SE coef	*T*‐value	*p*	VIF
Constant	23.83	0.00	*	*	
pH	1.538	0.000	*	*	1.00
Glucose	1.090	0.000	*	*	1.00
Asparagine	2.032	0.000	*	*	1.00
pH × pH	−1.232	0.000	*	*	1.01
Glucose × Glucose	0.006667	0.000000	*	*	1.01
Asparagine × Asparagine	0.2717	0.0000	*	*	1.01
pH × Glucose	0.000000	0.000000	*	*	1.00
pH × Asparagine	0.000000	0.000000	*	*	1.00
Glucose × Asparagine	0.000000	0.000000	*	*	1.00

*Note:* pH stands for “potential of hydrogen,” a measure of acidity or alkalinity. Horizontal titles: Coef = coefficient, SE Coef = standard error of coefficient, *T*‐value = *T*‐statistic value, *p* = probability, VIF = variance inflation factor. The star (*) denotes values that are statistically significant or placeholders for omitted data.

**TABLE 12:** Response surface regression: result versus pH; Glucose; Asparagine.

Asparagine	Glucose	pH
7.5	1	5
7.5	1	7
7.5	4	5
7.5	4	7
5	2.5	5
5	2.5	7
10	2.5	5
10	2.5	7
5	1	6
5	4	6
10	1	6
10	4	6
7.5	2.5	6
7.5	2.5	6
7.5	2.5	6

The *R* ^2^ value was found to be 100%, indicating an excellent fit between the experimental values and the predicted values. This is supported by the statistical data presented in Table 13, which shows the model summary including *R* ^2^, adjusted *R* ^2^, and other relevant statistics.

**TABLE 13:** Regression ANOVA for RSM of asparaginase production.

Statistic	Value	Source	DF	Adj SS	Adj MS	*F*‐value	*p*
		Model	9	67.5790	7.5088	56.31	0.000
*R* ^2^	1.000	Linear	3	61.4579	20.4860	153.55	0.000
Adjusted *R* ^2^	0.998	pH	1	18.9318	18.9318	141.86	0.000
Predicted *R* ^2^	0.995	Glucose	1	9.5048	9.5048	71.21	0.000
Standard deviation	0.15	Asparagine	1	33.0214	33.0214	247.57	0.000
Mean of response	72.5	Square	3	6.1210	2.0403	15.29	0.003
		pH*pH	1	5.6012	5.6012	41.98	0.001
		Glucose*Glucose	1	0.0002	0.0002	0.002	0.968
		Asparagine*Asparagine	1	0.2725	0.2725	2.04	0.215
		2‐Way Interaction	3	0.0000	0.0000	0.000	1.000
		pH*Glucose	1	0.0000	0.0000	0.000	1.000
		pH*Asparagine	1	0.0000	0.0000	0.000	1.000
		Glucose*Asparagine	1	0.0000	0.0000	0.000	1.000
		Error	5	0.0000	0.0000		
		Lack‐of‐Fit	3	0.0000	0.0000	*	*
		Pure Error	2	0.0000	0.0000		
		Total	14	67.5790			

*Note:* pH stands for “potential of Hydrogen,” a measure of acidity or alkalinity. Horizontal titles: DF = degrees of freedom, Adj SS = adjusted sum of squares, Adj MS = adjusted mean square, *F*‐value = *F*‐statistic value, *p* = probability. The star (*) denotes values that are statistically significant or placeholders for omitted data.

**FIGURE 11:**
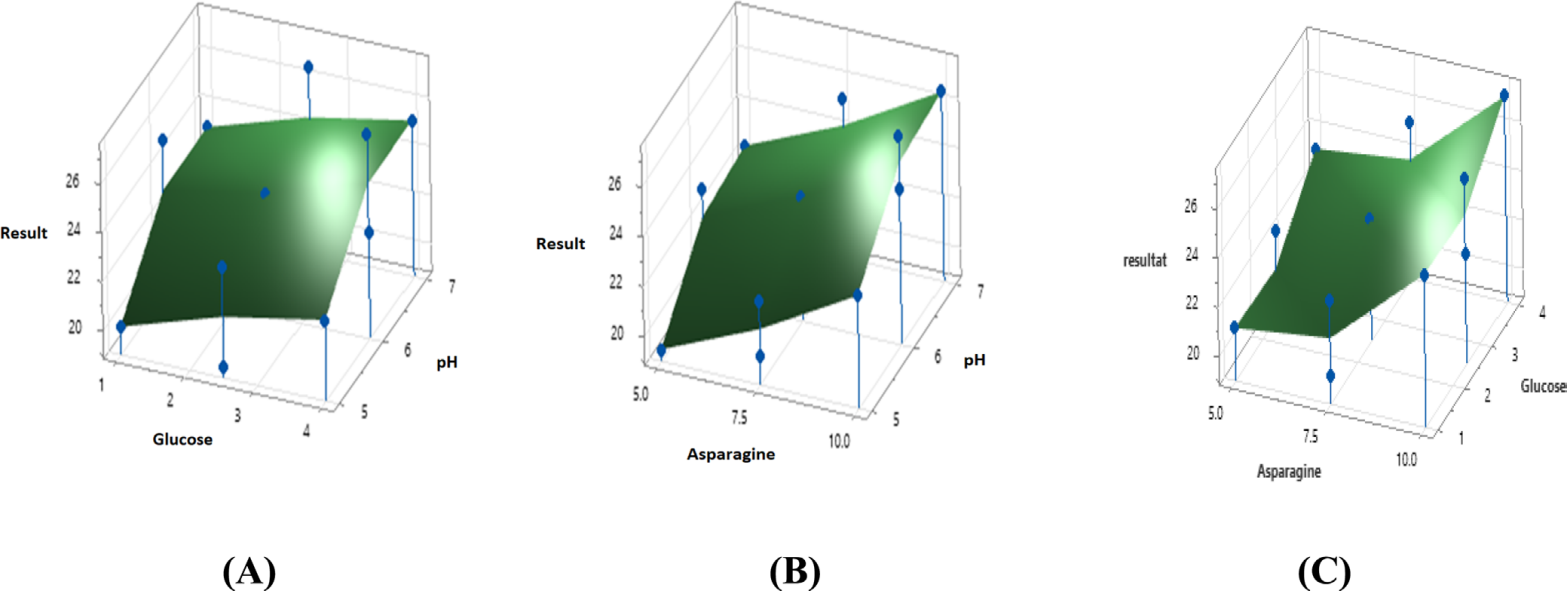
Three‐dimensional response surface plots showing the effect of Asparagine, pH, and Glucose and their mutual effect on the production of L‐asparaginase. (A) Surface plot of result versus pH, Glucose. (B) Surface plot of result versus pH, Asparagine. (C) Surface plot of result versus Glucose, Asparagine.

Figure 11 shows three‐dimensional response surface plots depicting the effect of asparagine, pH, and glucose on L‐asparaginase production. All three plots show that L‐asparaginase production increases with increasing asparagine concentration. This suggests that asparagine is a crucial nutrient for L‐asparaginase production. The effect of glucose concentration is more complex and varies depending on the pH and asparagine concentration. Plot A (pH vs. Glucose): At a constant asparagine concentration (5 g/L), L‐asparaginase production increases with increasing pH up to around 7.5, then decreases. The effect of glucose is again variable, with higher production. Plot B (Asparagine vs. pH): L‐asparaginase production is highest at around pH 7.5 and an asparagine concentration of 5 g/L. Higher or lower pH values or higher asparagine concentrations decrease production. Plot C (Asparagine vs. Glucose): At a constant pH (7.5), L‐asparaginase production increases with increasing asparagine concentration up to 5 g/L, then decreases. The effect of glucose is variable, with higher production. However, it is noteworthy that while the optimal pH for L‐asparaginase production is stated as 7.5, the maximum pH observed in Figure 11 is only 7. This discrepancy suggests that while enzyme activity is highest at a pH of 7, the optimal production conditions might extend slightly beyond this value.

These findings are in agreement with previous reports in the literature. For example, Yap et al. (2021) reported that asparagine concentration is a key limiting factor for L‐asparaginase production by Fusarium species. Furthermore, Kumar Meghavarnam and Janakiraman (2015) showed that the carbon source (glucose) plays a complex role in enzyme regulation, depending on its concentration and the fungal strain. Similarly, Uzma et al. (2016) demonstrated that L‐asparaginase production is enhanced under neutral to slightly alkaline pH conditions, confirming the optimal pH trend observed in our study. Our results are thus consistent with existing knowledge and contribute further insights into the optimization of L‐asparaginase production using endophytic *Fusarium* sp^3^.

## 4 Conclusions

The present study highlights the significant potential of fungal endophyte species—particularly those from the *Fusarium* genus—as a sustainable and efficient source for producing the anti‐cancer enzyme L‐asparaginase. This enzyme is widely used in the treatment of acute lymphoblastic leukemia due  to its ability to hydrolyze L‐asparagine, an essential amino acid for tumor cell proliferation. By systematically optimizing key fermentation parameters, including glucose concentration (optimal at 0.4%), pH (optimal at 7.0), and nitrogen source (asparagine), we achieved a maximum L‐asparaginase activity of 63.68 U/mL; underscoring the effectiveness of our approach.

Beyond its therapeutic applications, L‐asparaginase also holds substantial promise for use in the food industry, where it can play a critical role in reducing acrylamide formation, a toxic compound generated during the high‐temperature cooking of starchy foods such as potato chips, bread, and coffee. The enzyme achieves this by depleting L‐asparagine levels before thermal processing, thereby minimizing acrylamide generation. As such, the development of a reliable, high‐yield, and fungal‐based production method for L‐asparaginase presents a significant advancement toward safer food processing techniques.

Furthermore, this study demonstrates the utility of the Plackett–Burman design in identifying critical variables affecting enzyme production, which can serve as a foundation for further optimization. Future research will focus on scaling up the production process and integrating L‐asparaginase into commercial food safety protocols, enhancing its impact across both healthcare and food sectors. These findings affirm the broader biotechnological relevance of fungal endophytes and support continued exploration into their industrial applications.

## Author Contributions

**Wassima Lakhdari:** conceptualization (equal), data curation (equal), formal analysis (equal), funding acquisition (equal), investigation (equal), methodology (equal), project administration (equal), resources (equal), software (equal), supervision (equal), validation (equal), visualization (equal), writing – original draft (equal), writing – review and editing (equal). **Salah Neghmouche Nacer:** conceptualization (equal), data curation (equal), formal analysis (equal), funding acquisition (equal), investigation (equal), methodology (equal), project administration (equal), resources (equal), software (equal), supervision (equal), validation (equal), visualization (equal), writing – review and editing (equal). **Ibtissem Benyahia:** conceptualization (equal), resources (equal), software (equal). **Hamida Hammi:** conceptualization (equal), resources (equal), software (equal). **Hakim Bachir:** data curation (equal), resources (equal). **Djawahir Mouhoubi:** conceptualization (equal). **Yasmine Lakhdari:** conceptualization (equal), writing – review and editing (equal). **Ihsane Guemmou:** formal analysis (equal), writing – original draft (equal). **Abderrahmene Dehliz:** supervision (equal), validation (equal). **Barbara Sawicka:** conceptualization (equal), data curation (equal), formal analysis (equal), validation (equal), writing – original draft (equal), writing – review and editing (equal). **Sheikh F. Ahmad:** conceptualization (equal), data curation (equal), formal analysis (equal), funding acquisition (equal), investigation (equal), methodology (equal), resources (equal), software (equal), validation (equal), visualization (equal), writing – original draft (equal), writing – review and editing (equal). **Sabry M. Attia:** conceptualization (equal), funding acquisition (equal), methodology (equal), project administration (equal), validation (equal), visualization (equal), writing – original draft (equal), writing – review and editing (equal). **Amar Djemoui:** formal analysis (equal), resources (equal). **Mohammed Messaoudi:** conceptualization (equal), data curation (equal), formal analysis (equal), funding acquisition (equal), investigation (equal), methodology (equal), resources (equal), software (equal), supervision (equal), validation (equal), visualization (equal), writing – original draft (equal), writing – review and editing (equal).

## Ethics Statement

The authors have nothing to report.

## Conflicts of Interest

The authors declare no conflicts of interest.

## Data Availability

The data that support the findings of this study are available from the corresponding author upon reasonable request.
